# Clinical, Pathological, and Antimicrobial Resistance Features of *Staphylococcus aureus* Infections in Rabbits Raised Under Extensive Traditional Systems in Western Romania

**DOI:** 10.3390/vetsci13050466

**Published:** 2026-05-11

**Authors:** Vlad Iorgoni, Livia Stanga, Paula Nistor, Alexandru Gligor, Janos Degi, Bogdan Florea, Razvan Grigore Cojocaru, Ionica Iancu, Cosmin Horatiu Maris, Ioan Cristian Dreghiciu, Viorel Herman

**Affiliations:** 1 Department of Infectious Diseases and Preventive Medicine, Faculty of Veterinary Medicine, University of Life Sciences “King Mihai I” from Timişoara, 300645 Timişoara, Romania; vlad.iorgoni@usvt.ro (V.I.); paula.nistor@usvt.ro (P.N.); alexandru.gligor@usvt.ro (A.G.); janosdegi@usvt.ro (J.D.); ionica.iancu@usvt.ro (I.I.);; 2 Doctoral School “Veterinary Medicine”, University of Life Sciences “King Mihai I” from Timişoara, Calea Aradului 119, 300645 Timişoara, Romania; bogdan-alexandru.florea.fmv@usvt.ro; 3 Discipline of Microbiology, Faculty of Medicine, “Victor Babes” University of Medicine and Pharmacy, Eftimie Murgu Square 2, 300041 Timişoara, Romania; 4 Department of Internal Medicine, University of Life Sciences “King Mihai I” from Timişoara, 300645 Timişoara, Romania; 5 Department of Surgery, Faculty of Veterinary Medicine, University of Life Sciences “King Mihai I” from Timişoara, 300645 Timişoara, Romania; razvan.cojocaru@usvt.ro; 6 Department of Forestry, Faculty of Engineering and Applied Technologies, University of Life Sciences “King Mihai I” from Timișoara, 300645 Timișoara, Romania; cosmin.maris@usvt.ro; 7 Discipline of Parasitology, Faculty of Veterinary Medicine, University of Life Sciences “King Mihai I” from Timișoara, 300645 Timișoara, Romania; cristian.dreghiciu@usvt.ro; 8 Academy of Romanian Scientists (AOSR), Splaiul Independenței 54, 050094 Bucharest, Romania

**Keywords:** rabbit, *Staphylococcus aureus*, abscess, antimicrobial resistance, mastitis, pyometra, otitis, Romania

## Abstract

*Staphylococcus aureus* is a common opportunistic pathogen in rabbits and may cause localized or systemic infections that affect animal health and farm productivity. The present study aimed to investigate the clinical evolution, pathological lesions, and antimicrobial susceptibility profile of *S. aureus* infections in rabbits raised under traditional extensive systems in Western Romania. A total of 251 rabbits from 11 holdings located in Arad, Timiș, and Caraș-Severin counties were evaluated through epidemiological investigation, clinical examination, necropsy, and bacteriological analysis. Samples were cultured on Brain Heart Infusion medium and 5% sheep blood agar, and isolates were identified using matrix-assisted laser desorption/ionization time-of-flight mass spectrometry (MALDI-TOF MS). Antimicrobial susceptibility testing was performed using an automated system (VITEK 2, bioMérieux) and interpreted according to EUCAST guidelines. Among the examined animals, 68 rabbits (27.1%) showed clinical lesions compatible with *S. aureus* infection. The most common manifestations included subcutaneous abscesses, otitis externa, rhinitis, mammary abscesses, pyometra, and dental abscesses. Necropsy revealed suppurative and septicemic lesions affecting multiple organs. Antimicrobial susceptibility testing indicated high resistance to penicillin (100%), tetracycline (76.5%), doxycycline (67.6%), and amoxicillin (63.2%). In contrast, florfenicol (69.1% susceptible), ciprofloxacin (61.8%), gentamicin (54.4%), and enrofloxacin (52.9%) showed better antimicrobial activity. The results confirm the clinical and microbiological relevance of S. aureus infections in rabbits raised under traditional conditions and highlight the need for improved biosecurity measures and rational antimicrobial use.

## Simple Summary

*Staphylococcus aureus* is an important bacterial pathogen affecting rabbits and can cause a wide range of diseases, including skin abscesses, ear infections, respiratory disease, mastitis, and systemic infections. Such infections can lead to significant economic losses and welfare problems on rabbit farms. This study investigated the occurrence of *S. aureus* infections in rabbits raised in traditional extensive farming systems in Western Romania. A total of 251 rabbits from 11 holdings were clinically examined, and samples from affected animals were analyzed through bacteriological and pathological investigations. The most common lesions were subcutaneous abscesses, followed by otitis externa and rhinitis. Females were more frequently affected than males. Antimicrobial susceptibility testing revealed high resistance to several commonly used antibiotics, particularly penicillin and tetracycline. However, better activity was observed for florfenicol, ciprofloxacin, gentamicin, and enrofloxacin. These results highlight the importance of improved farm hygiene, early diagnosis, and responsible antimicrobial use in rabbit production systems.

## 1. Introduction

Rabbit production represents a relevant component of small-scale and backyard livestock systems across Europe, particularly in Eastern regions where traditional extensive farming practices remain prevalent. These systems play an important socio-economic role by supporting rural livelihoods, ensuring local protein sources, and maintaining genetic diversity. However, they are frequently associated with suboptimal biosecurity, inconsistent veterinary oversight, and heterogeneous management practices, all of which may facilitate the persistence and transmission of infectious agents [1,2].

Within this context, *Staphylococcus aureus* has emerged as a major opportunistic pathogen in rabbits, capable of causing a broad spectrum of clinical conditions ranging from localized infections to life-threatening systemic disease. Although it is commonly present as part of the normal microbiota of the skin and mucous membranes, its transition to pathogenicity is strongly influenced by host-related and environmental factors, including stress, trauma, overcrowding, and immunosuppression [1,3].

The clinical significance of *S. aureus* in rabbits is underscored by its association with multiple disease entities, including subcutaneous abscesses, pododermatitis, mastitis, otitis externa, rhinitis, pneumonia, reproductive disorders, and septicemia. Among these, abscess formation represents a defining feature of rabbit staphylococcosis, characterized by thick fibrous encapsulation and caseous necrotic material, which significantly impairs antimicrobial penetration and contributes to chronicity and recurrence [3,4].

The pathogenic success of *S. aureus* is largely attributable to its complex virulence arsenal, encompassing adhesins, cytotoxins, immune evasion factors, and enzymes that promote tissue invasion. In addition, its ability to form biofilms plays a critical role in persistent infections, enhancing bacterial survival under adverse conditions and increasing tolerance to antimicrobial agents [4,5,6,7].

From a therapeutic perspective, the growing prevalence of antimicrobial resistance among *S. aureus* strains represents a major challenge. Resistance to β-lactams and tetracyclines is widely documented, while the emergence of multidrug-resistant strains, including methicillin-resistant *S. aureus* (MRSA), raises concerns not only for animal health but also for public health due to the zoonotic potential of certain lineages [5,7,8,9].

Recent advances in diagnostic microbiology, particularly the implementation of matrix-assisted laser desorption/ionization time-of-flight mass spectrometry (MALDI-TOF MS), have significantly improved the accuracy, speed, and cost-effectiveness of bacterial identification. Similarly, automated antimicrobial susceptibility testing systems such as VITEK 2 provide standardized and reproducible resistance profiles, facilitating evidence-based therapeutic decisions and surveillance of antimicrobial resistance trends [10,11,12,13].

Despite the recognized importance of *S. aureus* infections in rabbits, most available studies have primarily focused on intensive commercial production systems, while data from traditional extensive farming environments remain limited, particularly in Eastern Europe. These systems are characterized by heterogeneous management practices, variable biosecurity levels, and increased environmental exposure, which may influence both disease dynamics and antimicrobial resistance patterns.

The novelty of the present study resides in its integrated clinical, pathological, and microbiological approach applied to rabbits raised under traditional extensive systems in Western Romania. Furthermore, this study provides region-specific data on the antimicrobial resistance profiles of *S. aureus* isolates, contributing to the limited epidemiological information available from this type of production system. By situating the findings within the broader European context, the study offers additional insight into the role of small-scale farming systems as potential reservoirs of antimicrobial-resistant pathogens within a One Health framework.

## 2. Materials and Methods

### 2.1. Study Area and Rabbit Holdings

**Figure 1:**
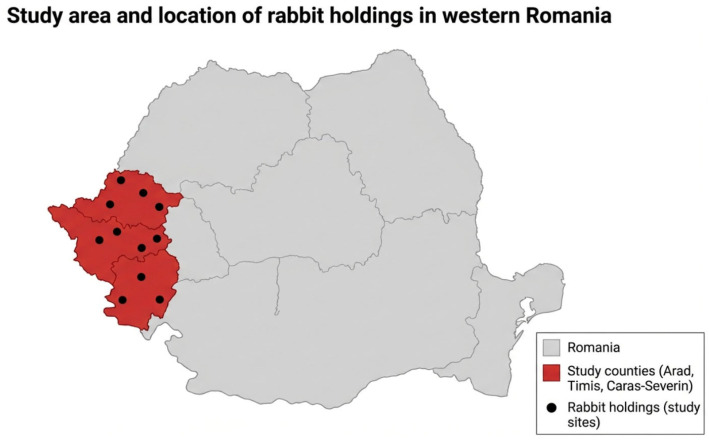
Geographic location of rabbit holdings included in the study conducted in Western Romania. The investigated farms were located in Arad, Timiș, and Caraș-Severin counties.

The study was conducted in eleven rabbit holdings located in Western Romania, specifically in Arad, Timiș, and Caraș-Severin counties (Figure 1). All farms used traditional extensive management systems.

The included holdings were selected based on the willingness of the owners to participate in the study, the presence of rabbits raised under traditional extensive conditions, and the feasibility of performing clinical examinations and sample collection.

Management practices varied between farms but were generally characterized by low to moderate biosecurity levels, the absence of standardized vaccination programs, and limited veterinary supervision. Animals were typically housed in outdoor or semi-open enclosures, with variable stocking densities.

Feeding practices consisted of a mixed diet including commercial feed, grains, fresh forage, and household food residues. Water was provided ad libitum.

Five of the investigated holdings belonged to breeders who regularly participated in rabbit exhibitions, which may increase pathogen circulation due to animal movement and contact with external populations [14].

### 2.2. Animals

A total of 251 rabbits aged between two months and three years were included in the study. The animals belonged to multiple breeds, including German Spotted Giant, German Giant, Rex, Blanc de Pannonia, German Lop, Small Spotted, Dwarf Spotted, Loh Dwarf, Szekler rabbit, Lionhead, and mixed-breed rabbits.

The sex distribution included 74 males and 177 females.

All animals included in the study originated from privately owned rabbit holdings. Informed consent was obtained from all owners prior to clinical examination and sample collection.

The study did not involve experimental procedures, and all investigations were performed as part of routine veterinary diagnostic activities.

The study protocol was reviewed and approved by the Ethics Committee of the Faculty of Veterinary Medicine, University of Life Sciences “King Mihai I” from Timișoara (Permit No. 668/01.04.2026).

### 2.3. Clinical Examination

All animals underwent a detailed clinical examination performed by trained veterinarians. The evaluation focused on detecting dermatological, respiratory, mammary, and reproductive abnormalities [15].

Particular attention was given to identifying subcutaneous swellings suggestive of abscess formation, signs of otitis externa such as ear discharge and pruritus, respiratory symptoms including nasal discharge and sneezing, and reproductive disorders such as mammary gland inflammation or abnormal genital discharge.

General clinical parameters, including body condition, behavior, and signs of systemic illness, were also recorded.

### 2.4. Sample Collection

Biological samples were collected from animals presenting clinical signs suggestive of bacterial infection, as well as from rabbits that died during the study period. Sampling was performed using a convenience-based approach, targeting clinically affected individuals.

In most cases, one sample per lesion per animal was collected. However, in animals presenting lesions at multiple anatomical sites, multiple samples were obtained.

Sampling sites included abscess contents, nasal secretions, ear exudates, mammary lesions, and internal organs collected during necropsy, such as the lungs, liver, spleen, and kidneys. All samples were obtained using sterile techniques to avoid contamination and were transported under appropriate conditions to the laboratory for microbiological analysis [16].

### 2.5. Bacteriological Examination

Samples were inoculated onto Brain Heart Infusion (BHI) medium and 5% sheep blood agar and incubated aerobically at 37 °C for 24–48 h [17,18].

Aerobic culture conditions were selected because *Staphylococcus aureus* is a facultative anaerobic organism that grows readily under these conditions.

Although other bacterial agents may be involved in similar clinical conditions, the study focused on *Staphylococcus aureus* as the primary pathogen isolated from the investigated lesions.

Following incubation, bacterial growth was evaluated based on colony morphology, hemolytic activity, and Gram staining characteristics. Colonies suggestive of *Staphylococcus* spp. were further analyzed.

*Staphylococcus aureus* was predominantly isolated in monoculture; however, in some cases, mixed bacterial populations were also observed.

Definitive identification of isolates was performed using MALDI-TOF MS, a rapid and highly accurate technique based on protein spectral analysis. This method allows reliable species-level identification and has become a reference standard in modern microbiology laboratories [10,12,13].

### 2.6. Antimicrobial Susceptibility Testing

Antimicrobial susceptibility testing was carried out using the automated VITEK 2 system (bioMérieux), employing standardized cards designed for Gram-positive bacteria [11].

Interpretation of antimicrobial susceptibility results was performed according to EUCAST guidelines, which are widely adopted in European clinical microbiology laboratories and ensure consistency with regional antimicrobial resistance surveillance.

The VITEK 2 system (bioMérieux) was used according to the manufacturer’s instructions, and results were interpreted using standardized breakpoints to classify isolates into susceptible and resistant categories [11].

The antimicrobial panel included agents frequently used in veterinary medicine or commonly included in antimicrobial susceptibility testing panels for Gram-positive bacteria agents in veterinary practice, such as amoxicillin, ciprofloxacin, doxycycline, enrofloxacin, florfenicol, gentamicin, neomycin, trimethoprim/sulfamethoxazole, tetracycline, and penicillin [19,20].

The antimicrobial agents included in the susceptibility testing panel and their interpretation categories are presented in Table 1.

### 2.7. Pathological Examination

Complete necropsies were performed on rabbits that died during the study. Macroscopic examination focused on identifying lesions indicative of suppurative or systemic infection [21].

### 2.8. Statistical Analysis

Data were analyzed using descriptive statistical methods. Absolute frequencies and percentages were calculated to summarize the distribution of clinical findings and antimicrobial resistance patterns. The overall prevalence of rabbits presenting lesions compatible with *Staphylococcus aureus* infection was estimated together with 95% confidence intervals (95% CI). In addition, sex-specific and farm-type-specific prevalence values were calculated with corresponding 95% confidence intervals.

Comparisons between categorical variables were performed using the Chi-square (χ^2^) test. The associations between sex and the occurrence of clinical lesions, as well as between exhibition participation (exhibition-related farm status) and lesion occurrence, were evaluated using this test. Degrees of freedom (df) and *p*-values were reported, and a *p*-value of less than 0.05 was considered statistically significant. Furthermore, odds ratios (OR) with 95% confidence intervals were calculated to estimate the strength of association between exhibition-related farm status and the occurrence of clinical lesions.

Clinical lesion categories were expressed as proportions of affected animals (*n* = 68), with corresponding 95% confidence intervals. Because multiple lesions could be recorded in the same animal, lesion categories were considered non-mutually exclusive and were analyzed descriptively.

Given the convenience-based sampling strategy, inferential statistical analyses were interpreted cautiously and used only for exploratory purposes. All results were therefore considered within the descriptive and observational scope of the study.

## 3. Results

### 3.1. Clinical Findings

Among the 251 examined rabbits, 68 animals presented lesions compatible with *Staphylococcus aureus* infection, corresponding to an overall prevalence of 27.1% (95% CI: 22.0–32.9%). Of the 177 females included in the study, 48 were affected (27.1%; 95% CI: 21.1–34.1%), while 20 of the 74 males were affected (27.0%; 95% CI: 18.2–38.1%). No statistically significant association was identified between sex and the occurrence of clinical lesions (χ^2^ = 0.0002, df = 1, *p* = 0.988).

When holdings were stratified according to exhibition participation, 43 of 132 rabbits from exhibition farms presented lesions compatible with infection (32.6%; 95% CI: 25.2–41.0%), whereas 25 of 119 rabbits from non-exhibition farms were affected (21.0%; 95% CI: 14.7–29.2%). This difference was statistically significant (χ^2^ = 4.24, df = 1, *p* = 0.039). Furthermore, rabbits originating from exhibition farms showed higher odds of lesion occurrence compared to those from non-exhibition farms (OR = 1.82, 95% CI: 1.03–3.22).

**Figure 2:**
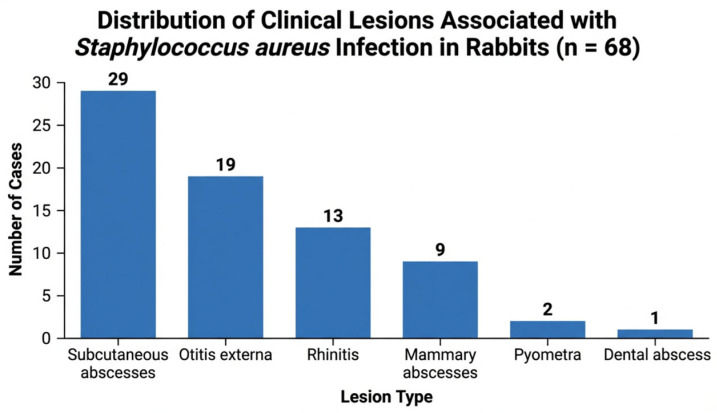
Distribution of clinical lesions associated with *Staphylococcus aureus* infection in rabbits (*n* = 68). Values represent the number of cases for each lesion category. Because multiple lesions could be present in the same animal, categories are not mutually exclusive.

The most frequent lesions included subcutaneous abscesses, otitis externa, rhinitis, mammary abscesses, pyometra, and dental abscesses. The distribution of clinical lesions is presented in Figure 2.

Among the 68 affected rabbits, subcutaneous abscesses were the most frequent lesion, being identified in 29 cases (42.6%; 95% CI: 31.3–54.8%), followed by otitis externa in 19 cases (27.9%; 95% CI: 18.6–39.6%) and rhinitis in 13 cases (19.1%; 95% CI: 11.4–29.8%). Mammary abscesses were recorded in 9 cases (13.2%; 95% CI: 7.1–23.5%), while less frequent lesions included pyometra (2 cases; 2.9%; 95% CI: 0.8–10.0%) and dental abscesses (1 case; 1.5%; 95% CI: 0.3–7.9%).

Subcutaneous abscesses were the most common lesion, followed by otitis externa and rhinitis, while reproductive and dental lesions were less frequently observed. Mammary abscesses were observed mainly in lactating females and often involved multiple mammary glands. Otitis externa manifested as pruritus, ear discharge, and occasionally head tilt. Rhinitis was characterized by mucopurulent nasal discharge and sneezing.

### 3.2. Necropsy Findings

Necropsy examination revealed severe suppurative lesions consistent with septicemic infection. The most prominent findings included pulmonary congestion and bronchopneumonia, frequently accompanied by pulmonary abscesses and pleuritis with hydrothorax. Abdominal lesions were characterized by fibrinous peritonitis, hepatic congestion with focal abscess formation, splenomegaly, and varying degrees of renal degeneration. In female rabbits, reproductive tract involvement was also observed, including metritis and pyometra.

No formal scoring system was applied for lesion severity; clinical findings were recorded descriptively based on the presence and anatomical distribution of lesions.

### 3.3. Antimicrobial Susceptibility

The antimicrobial susceptibility profiles revealed a concerning pattern of resistance, particularly to penicillin, tetracycline, doxycycline, and amoxicillin, indicating widespread reduced efficacy of commonly used first-line antimicrobial agents.

This resistance pattern suggests prolonged or inappropriate antimicrobial exposure under field conditions, which may have contributed to selective pressure and the emergence of resistant strains. In contrast, higher susceptibility rates were observed for florfenicol, ciprofloxacin, gentamicin, and enrofloxacin, indicating that these agents may still retain therapeutic value in clinical practice.

**Figure 3:**
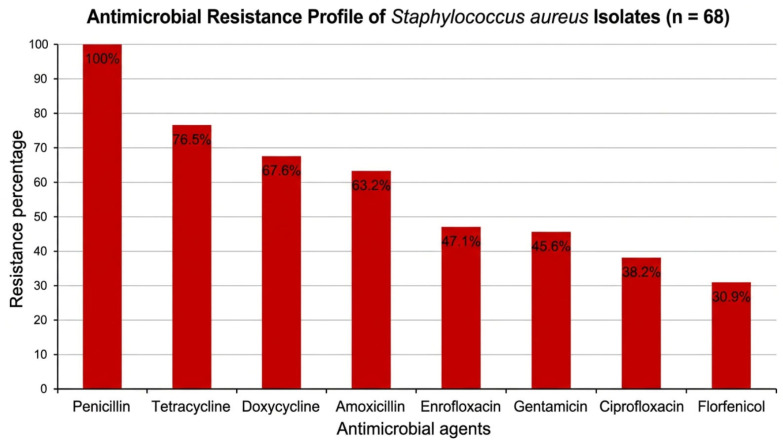
Antimicrobial resistance profile of *Staphylococcus aureus* isolates recovered from rabbits (*n* = 68). High resistance levels were observed for penicillin, tetracycline, doxycycline, and amoxicillin, while lower resistance rates were recorded for fluoroquinolones and florfenicol.

However, the variability in susceptibility highlights the necessity of performing antimicrobial susceptibility testing prior to treatment whenever possible, rather than relying on empirical therapy. The antimicrobial resistance profile of the isolates is shown in Figure 3.

The antimicrobial susceptibility profiles revealed high levels of resistance to several commonly used antimicrobial agents. Resistance to penicillin was observed in 100% of isolates (95% CI: 94.7–100%), followed by tetracycline (76.5%; 95% CI: 65.0–85.5%), doxycycline (67.6%; 95% CI: 55.7–77.6%), and amoxicillin (63.2%; 95% CI: 51.3–73.7%).

In contrast, higher susceptibility rates were recorded for florfenicol (69.1% susceptible; 95% CI: 57.3–79.0%), ciprofloxacin (61.8%; 95% CI: 49.9–72.5%), gentamicin (54.4%; 95% CI: 42.6–65.7%), and enrofloxacin (52.9%; 95% CI: 41.1–64.4%).

These findings indicate substantial variability in antimicrobial efficacy and highlight the importance of performing susceptibility testing prior to treatment.

## 4. Discussion

The present study provides insights into the clinical, pathological, and microbiological characteristics of *Staphylococcus aureus* infections in rabbits raised under traditional extensive farming conditions.

The relatively high prevalence of clinically affected animals observed in this study supports the hypothesis that traditional farming systems, although less intensive, may still provide favorable conditions for the maintenance and transmission of opportunistic pathogens. Factors such as variable hygiene standards, environmental exposure, and limited disease monitoring likely contribute to this epidemiological scenario [2,3].

The higher occurrence observed in exhibition-associated holdings may reflect the epidemiological impact of animal movement, increased inter-herd contact, transport-related stress, and greater opportunities for pathogen circulation. In the present study, rabbits from exhibition farms showed significantly higher odds of lesion occurrence than rabbits from non-exhibition holdings. Although this association should be interpreted cautiously in light of the convenience-based design, it supports the hypothesis that exhibition-related management may contribute to the transmission and persistence of *Staphylococcus aureus* in traditional rabbit production systems.

The predominance of subcutaneous abscesses aligns with the well-documented pathophysiology of staphylococcal infections in rabbits. The encapsulated structure of these lesions, combined with the production of biofilms, creates a microenvironment that protects bacteria from both host immune responses and antimicrobial agents, thereby explaining the chronic and recurrent nature of the disease [21,22].

However, the sex-specific prevalence values were nearly identical in the present dataset, and the Chi-square analysis did not support a statistically significant association between sex and lesion occurrence. Therefore, any apparent predominance of female cases should be interpreted with caution and may reflect the underlying sex distribution of the study population rather than a true biological predisposition. Lactation-related stress, mammary gland susceptibility, and reproductive tract exposure may all contribute to increased vulnerability to infection. However, this observation should be interpreted with caution, as no statistically significant differences were identified [23,24,25].

The identification of systemic lesions in necropsied animals indicates that *S. aureus* infections may progress beyond localized disease, leading to septicemia and multiorgan involvement. This progression is particularly relevant in cases where diagnosis and treatment are delayed or inadequate [26,27].

A major strength of the present study is the use of advanced diagnostic techniques, including MALDI-TOF MS for bacterial identification and the VITEK 2 system for antimicrobial susceptibility testing. These methods provide high accuracy, reproducibility, and rapid turnaround times, representing a significant improvement over conventional phenotypic approaches. Their implementation in veterinary diagnostics contributes to more reliable data and supports antimicrobial stewardship [10,11,12,13].

The antimicrobial resistance patterns observed in this study are consistent with those reported in other European settings, particularly the high resistance rates to β-lactams and tetracyclines. These findings raise concerns regarding the effectiveness of commonly used treatments and highlight the need for prudent antimicrobial use [3,28,29,30,31].

From a One Health perspective, the presence of resistant *S. aureus* strains in rabbit populations is of particular importance. Livestock-associated strains may act as reservoirs of resistance genes, with potential transmission to humans through direct contact or environmental pathways [3,32,33,34,35]. Although molecular characterization was not performed in this study, the observed resistance profiles underscore the importance of continued surveillance.

The antimicrobial resistance patterns observed in this study may be influenced by farm-level factors, including empirical antimicrobial use, limited veterinary supervision, and inconsistent treatment protocols. These conditions can contribute to selective pressure and the emergence of resistant strains [32,33,36,37].

From a One Health perspective, traditional rabbit farming systems may represent potential reservoirs of antimicrobial-resistant *Staphylococcus aureus*, with possible transmission between animals, the environment, and humans [3,38,39,40].

From a clinical perspective, the findings of the present study have important implications for veterinary practice in small-scale and backyard rabbit production systems. The high prevalence of suppurative lesions, particularly subcutaneous abscesses, highlights the need for early detection and prompt intervention in order to prevent disease progression and systemic dissemination.

The observed antimicrobial resistance patterns emphasize the necessity of performing antimicrobial susceptibility testing prior to treatment whenever possible, rather than relying on empirical therapy. The reduced effectiveness of commonly used antimicrobials, such as penicillin and tetracyclines, may lead to therapeutic failure if not properly addressed.

In addition, the results underline the importance of improving basic management practices, including hygiene, housing conditions, and biosecurity measures, as key components in reducing the incidence and recurrence of staphylococcal infections. Veterinary guidance and targeted education for breeders are essential to promote responsible antimicrobial use and to limit the emergence and spread of resistant strains in traditional farming systems.

### Limitations

The study has several limitations that should be acknowledged. Molecular characterization of isolates, including the detection of resistance genes or virulence factors, was not performed, which limits the ability to assess clonal relationships or zoonotic potential. In addition, the convenience-based sampling strategy, focused on clinically affected animals and diagnostic submissions, limits the generalizability of the findings to the broader rabbit population. For this reason, inferential statistical analyses should be interpreted cautiously. Furthermore, the study design did not include systematically collected individual- or farm-level exposure variables, which precluded the use of more advanced multivariable models for risk factor analysis.

## 5. Conclusions

*Staphylococcus aureus* infections represent a significant and multifactorial health problem in rabbits raised under traditional extensive farming systems.

The disease is characterized by diverse clinical manifestations, with a predominance of subcutaneous abscesses, but also including respiratory, reproductive, and systemic involvement. The observed antimicrobial resistance patterns, particularly the high resistance to commonly used antibiotics, emphasize the urgent need for improved antimicrobial stewardship.

The application of advanced diagnostic tools such as MALDI-TOF MS and automated susceptibility testing systems enhances diagnostic accuracy and should be encouraged in veterinary practice.

From both veterinary and public health perspectives, the findings highlight the necessity of implementing improved hygiene measures, strengthening biosecurity, and promoting responsible antimicrobial use within a One Health framework.

## Figures and Tables

**Table 1:** Antimicrobials included in susceptibility testing and interpretation categories.

Antimicrobial	Abbreviation	Interpretation Categories
Penicillin	PEN	S/R
Amoxicillin	AML	S/R
Tetracycline	TE	S/R
Doxycycline	DOX	S/R
Enrofloxacin	ENR	S/R
Ciprofloxacin	CIP	S/R
Gentamicin	GEN	S/R
Florfenicol	FFC	S/R
Trimethoprim/sulfamethoxazole	SXT	S/R
Neomycin	NEO	S/R

## Data Availability

The original contributions presented in this study are included in the article. Further inquiries can be directed to the corresponding author.

## Abbreviations

The following abbreviations are used in this manuscript:

AML	Amoxicillin
BHI	Brain Heart Infusion
CIP	Ciprofloxacin
DOX	Doxycycline
ENR	Enrofloxacin
EUCAST	European Committee on Antimicrobial Susceptibility Testing
FFC	Florfenicol
GEN	Gentamicin
MRSA	Methicillin-resistant *Staphylococcus aureus*
NEO	Neomycin
PEN	Penicillin
SXT	Trimethoprim/Sulfamethoxazole
TE	Tetracycline
